# Middle East Respiratory Coronavirus Accessory Protein 4a Inhibits PKR-Mediated Antiviral Stress Responses

**DOI:** 10.1371/journal.ppat.1005982

**Published:** 2016-10-26

**Authors:** Huib H. Rabouw, Martijn A. Langereis, Robert C. M. Knaap, Tim J. Dalebout, Javier Canton, Isabel Sola, Luis Enjuanes, Peter J. Bredenbeek, Marjolein Kikkert, Raoul J. de Groot, Frank J. M. van Kuppeveld

**Affiliations:** 1 Virology Division, Department of Infectious Diseases and Immunology, Faculty of Veterinary Medicine, Utrecht University, Utrecht, The Netherlands; 2 Molecular Virology Laboratory, Department of Medical Microbiology, Leiden University Medical Center, Leiden, The Netherlands; 3 Department of Molecular and Cell Biology, National Center of Biotechnology (CNB-CSIC), Campus Universidad Autonoma de Madrid, Madrid, Spain; University of Maryland School of Medicine, UNITED STATES

## Abstract

Middle East respiratory syndrome coronavirus (MERS-CoV) causes severe respiratory infections that can be life-threatening. To establish an infection and spread, MERS-CoV, like most other viruses, must navigate through an intricate network of antiviral host responses. Besides the well-known type I interferon (IFN-α/β) response, the protein kinase R (PKR)-mediated stress response is being recognized as an important innate response pathway. Upon detecting viral dsRNA, PKR phosphorylates eIF2α, leading to the inhibition of cellular and viral translation and the formation of stress granules (SGs), which are increasingly recognized as platforms for antiviral signaling pathways. It is unknown whether cellular infection by MERS-CoV activates the stress response pathway or whether the virus has evolved strategies to suppress this infection-limiting pathway. Here, we show that cellular infection with MERS-CoV does not lead to the formation of SGs. By transiently expressing the MERS-CoV accessory proteins individually, we identified a role of protein 4a (p4a) in preventing activation of the stress response pathway. Expression of MERS-CoV p4a impeded dsRNA-mediated PKR activation, thereby rescuing translation inhibition and preventing SG formation. In contrast, p4a failed to suppress stress response pathway activation that is independent of PKR and dsRNA. MERS-CoV p4a is a dsRNA binding protein. Mutation of the dsRNA binding motif in p4a disrupted its PKR antagonistic activity. By inserting p4a in a picornavirus lacking its natural PKR antagonist, we showed that p4a exerts PKR antagonistic activity also under infection conditions. However, a recombinant MERS-CoV deficient in p4a expression still suppressed SG formation, indicating the expression of at least one other stress response antagonist. This virus also suppressed the dsRNA-independent stress response pathway. Thus, MERS-CoV interferes with antiviral stress responses using at least two different mechanisms, with p4a suppressing the PKR-dependent stress response pathway, probably by sequestering dsRNA. MERS-CoV p4a represents the first coronavirus stress response antagonist described.

## Author Summary

Human coronaviruses generally cause relatively mild respiratory disease. In the past 15 years, the world has witnessed the emergence of two coronaviruses with high mortality rates in humans; severe acute respiratory syndrome coronavirus (SARS-CoV) in 2002 and Middle East respiratory syndrome coronavirus (MERS-CoV) in 2012, both originating from animal reservoirs. Successful infection of a host not only depends on the presence of an appropriate receptor but also on the ability of a virus to evade innate antiviral host responses, which constitute the first line of defense against invading viruses. MERS-CoV has been reported to actively suppress the IFN-α/β response, but it is unknown whether it also interferes with another important innate antiviral response, the stress response pathway. Activation of this pathway by a kinase, PKR, curtails virus infection by shutting off cellular and viral protein synthesis. To date, no coronavirus protein has been recognized to suppress the stress response pathway. Here, we show that the accessory protein 4a of MERS-CoV is a potent stress antagonist that prevents PKR activation by sequestering its ligand, dsRNA. This finding furthers our understanding of the molecular mechanism used by MERS-CoV to evade infection-limiting antiviral host responses and may provide new avenues for therapeutic intervention.

## Introduction

Innate antiviral responses represent the first line of defense against invading viral pathogens. Host cells are equipped with multiple mechanisms to detect and respond to non-self, pathogen-associated molecular patterns (PAMPs). One of these PAMPs, viral cytosolic RNA, can be detected by RIG-I-like receptors (RLRs), such as melanoma differentiation-associated protein 5 (MDA5) and retinoic acid inducible gene 1 (RIG-I). Upon recognition of viral, non-self RNA, signal transduction pathways are activated, which results in the expression of type I interferons (IFN-α/β), proinflammatory cytokines and chemokines. Secreted IFN-α/β triggers the transcription of interferon-stimulated genes (ISGs), both in infected as neighboring cells, and thereby implements an antiviral state that restricts virus propagation in the host.

Growing evidence points to an important role of the stress response pathway as an additional innate antiviral response [1,2]. One of the ISGs, protein kinase R (PKR), detects viral RNA in the cytoplasm, which induces its autophosphorylation and subsequent phosphorylation of the alpha subunit of eukaryotic translation initiation factor 2 (eIF2α). PKR mediated phosphorylation of eIF2α inactivates (viral) protein synthesis, thereby affecting virus propagation. Stalled translation initiation complexes, together with nucleating factors like G3BP1, G3BP2, TIA-1 and many translation initiation factors like eIF3, form cytoplasmic aggregates, which are called stress granules (SGs). The role of these SGs remains controversial, but growing evidence points to a role of these SGs as a platform for antiviral signal transduction [3–5].

To ensure efficient virus replication, many viruses encode proteins with specialized functions to evade innate antiviral responses, although their mode of action and the point of interference may differ. Viruses usually interfere in several antiviral pathways and even disrupt pathways at multiple levels, to ensure efficient suppression of the host innate antiviral responses. A well-studied example is the Influenza A virus NS1 protein, which, among many other evasive functions, shields viral double-stranded RNA (dsRNA) from detection by both RLRs and PKR [6,7], thus blocking IFN-α/β and antiviral stress response pathways, respectively.

Coronaviruses are large positive-stranded RNA viruses belonging to the order *Nidovirales*. The coronavirus genome is typically between 26 and 32 kb in size and encodes more than 20 proteins. The 5’ open reading frame (ORF)1ab encodes the non-structural proteins (nsps), which together form the replication-transcription machinery. The 3’ end of the coronavirus genome contains several additional ORFs encoding the structural proteins and a varying number of accessory proteins. These accessory proteins often lack any detectable homology to other viral or host proteins and their function is unknown in many cases. A common feature, however, is that they are often not essential for virus replication *per se* but are important for virulence, suggesting that accessory proteins serve to modulate host antiviral responses [8–13].

Human coronaviruses generally cause mild respiratory symptoms. Exceptions are severe acute respiratory coronavirus (SARS-CoV), which emerged in China in 2002 through cross-species transmissions from bats and civet cats [14], and Middle East respiratory syndrome coronavirus (MERS-CoV), which emerged in the Arabian Peninsula in 2012. MERS-CoV causes acute and severe respiratory symptoms and continues to make a serious impact on the local as well as the global health system with over 1,694 laboratory confirmed cases and 605 deaths as of March 21^st^ 2016 [15]. This virus is believed to be transmitted to humans primarily via animal hosts, most likely dromedary camels [16,17]. As yet, little is known about how MERS-CoV modulates host antiviral responses. There is firm evidence that MERS-CoV inhibits IFN-α/β production [18–20] and several viral proteins have been implicated in this evasion mechanism–including accessory protein 4a (p4a), which is a dsRNA-binding protein [21–23]–but the inhibitory effect of these proteins on innate antiviral responses has thus far only been demonstrated in transfected cells expressing these viral proteins, not during virus infection. Whether MERS-CoV has also evolved mechanisms to modulate the stress response pathway is unknown thus far.

Here, we show for the first time that MERS-CoV actively suppresses the stress response pathway and we identify the accessory protein 4a as a potent inhibitor of the PKR-mediated stress response pathway. Furthermore, we provide evidence that the rescue of translation and inhibition of SG formation rely on p4a’s dsRNA-binding function, suggesting that it exerts antagonistic activity by sequestering dsRNA from recognition by PKR. Moreover, evidence for the existence of at least one other MERS-CoV encoded stress response antagonist is provided.

## Results

### MERS-CoV blocks stress responses in infected cells

**Fig 1:**
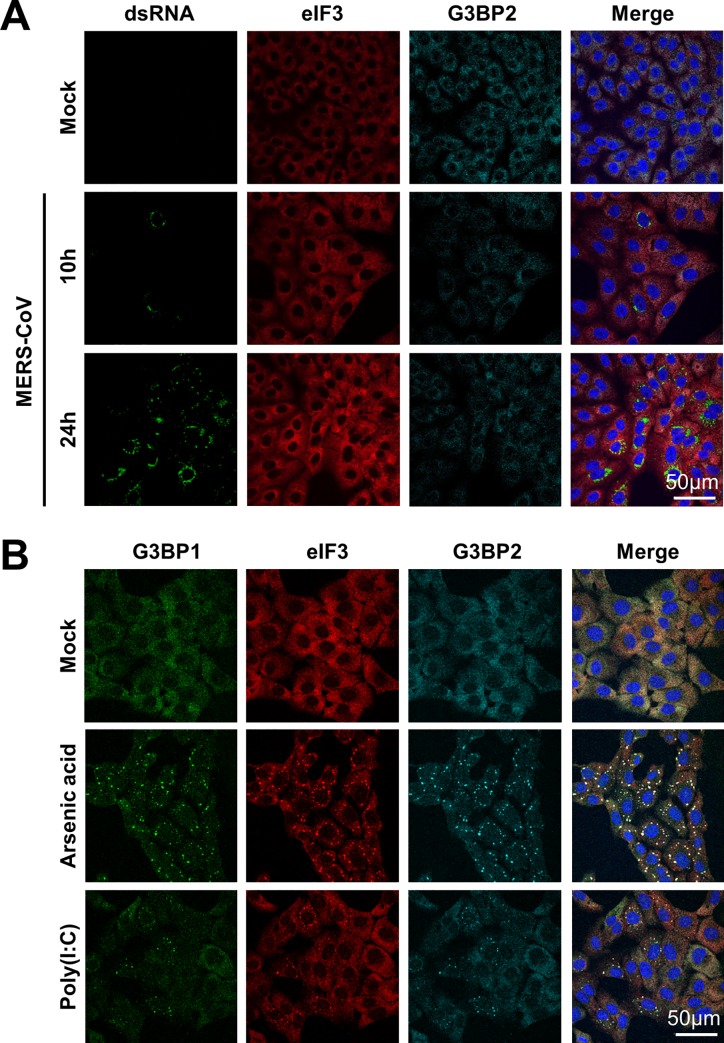
MERS-CoV infection fails to activate the stress response pathway. (A) Immune fluorescence images of mock-treated an MERS-CoV infected Vero cells. Cells were infected with an MOI of 1 and fixed using 3% paraformaldehyde in PBS at 10h or 24h post infection. Cells were stained for dsRNA, and stress granule markers eIF3 and G3BP2. (B) Immune fluorescence images of cells treated with arsenic acid (0.5 mM for 60 min) or transfected with poly(I:C) and stained for eIF3, G3BP1 and G3BP2.

To investigate whether MERS-CoV infection activates the stress response pathway, Vero cells were infected with MERS-CoV (MOI = 1) and analyzed for the occurrence of SG at regular time intervals by visualizing the subcellular localization of eIF3 and G3BP2, which are established markers for SGs. In parallel, the efficiency of virus infection was monitored by visualizing dsRNA using the J2 antibody. Despite efficient virus infection and replication, as indicated by the accumulation of considerable amounts of viral dsRNA in the cytosol, no SGs were observed at any of the indicated time points (Fig 1A). The lack of SGs was not due to an intrinsic defect in the stress response pathway of Vero cells as clear SGs were formed upon arsenic acid treatment and poly(I:C) transfection (Fig 1B). Together, these findings indicate that MERS-CoV either hides its viral RNA from detection by PKR, possibly through the formation of double membrane vesicles [24], and/or that it encodes one or more antagonists to suppress activation of the stress response pathway.

### MERS-CoV p4a suppresses dsRNA- and PKR-dependent formation of SGs

**Fig 2:**
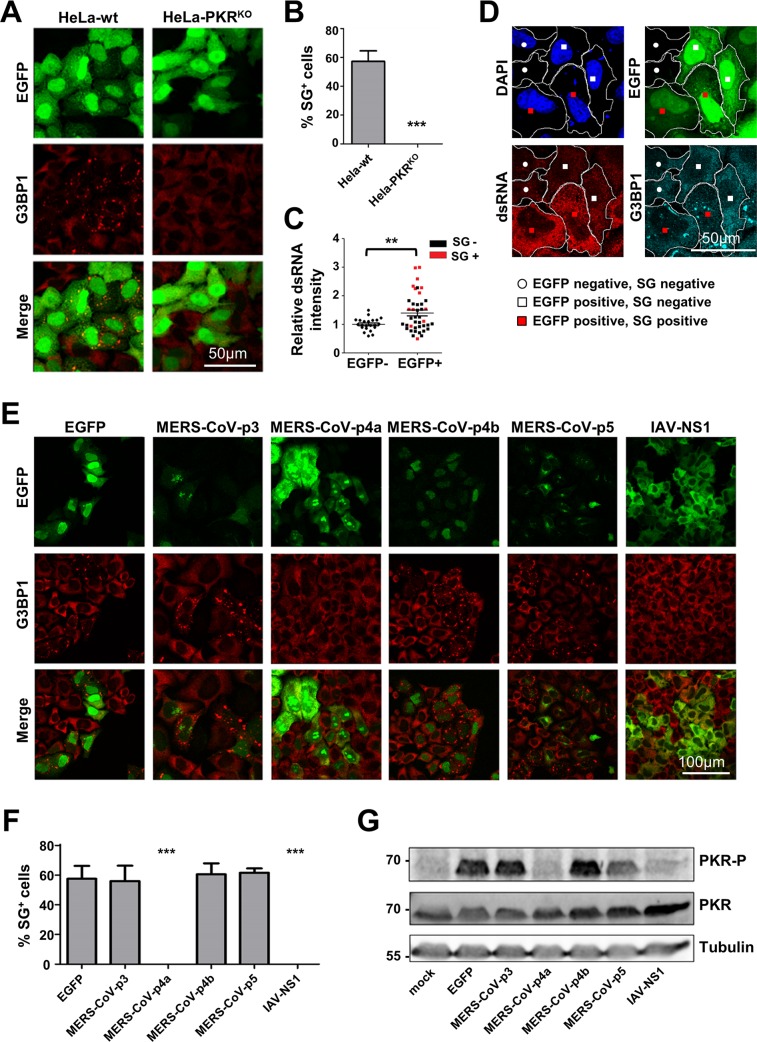
MERS-CoV p4a suppresses dsRNA-dependent and PKR-mediated stress in transfected cells. (A) Immune fluorescence images of HeLa-wt or HeLa-PKR^KO^ cells transfected with pEGFP-N3 plasmid (500 ng/well). Cells were fixed at 24h post transfection using paraformaldehyde and stained for G3BP1 (shown in red). EGFP expression is shown in green. (B) Quantification of SG-positive cells. SG-positive cells were quantified from three randomly selected images. Shown are means with standard deviations, analyzed using an unpaired t-test (***, p<0.001). (C) Quantification of the average dsRNA staining intensity in individual cells using imageJ software. Intensity levels are plotted relative to that of the non-transfected cells from the same images. Cells were classified as non-transfected or transfected based on EGFP expression, and as SG-positive or SG-negative based on presence of G3BP1 aggregates. Differences in relative dsRNA intensity levels were analyzed using an unpaired t-test (**, p<0.01). (D) Typical example of the IFA images used for quantification in C. Borders of two cells of each phenotype (EGFP-; EGFP+SG-; EGFP+SG+) are indicated in white. (E) Immune fluorescence images of HeLa cells transfected with pEGFP expression plasmids. Cells were fixed at 24h post transfection and stained for G3BP1 (shown in red). EGFP expression is shown in green. (F) Quantification of SG-positive cells. Analysis was performed as described in panel B (***, p<0.001). (G) Western blot analysis of PKR and phospho-PKR in HeLa cell lysates at 24h post pEGFP plasmid transfection. Tubulin expression was detected as loading control.

To investigate whether MERS-CoV accessory proteins can suppress the stress response pathway, we expressed them individually as EGFP fusion proteins and monitored SG formation in transfected cells. This approach is based on the observation that transfection of plasmid DNA, and in particular the pEGFP plasmids, can activate PKR, most likely due to the production of dsRNA formed from positive and negative sense mRNA transcription from cryptic promoters in these plasmids [25]. Indeed, we observed that transfection of pEGFP plasmid DNA in HeLa cells triggered SG formation in a PKR-dependent manner, as no SGs were observed in PKR knockout cells (HeLa-PKR^KO^), which we generated using the CRISPR-Cas9 system (S1 Fig) (Fig 2A and 2B). Also, using the J2 anti-dsRNA antibody, we noticed a significant increase in dsRNA levels in cells transfected with pEGFP plasmid DNA and especially in cells that displayed SGs (Fig 2C and 2D). This phenomenon was not restricted to the pEGFP plasmid as all plasmids with eukaryotic promoters induced SG formation in our HeLa cells, albeit to different levels, while those with prokaryotic promoters did not (S2 Fig). Together, these data support the idea that transfection of pEGFP plasmid DNA can trigger dsRNA-dependent and PKR-mediated SG formation, and provide the basis for a convenient and versatile method to test potential antagonistic activities of viral proteins by expressing them as EGFP fusion proteins.

Plasmids each encoding one of the four MERS-CoV accessory proteins fused to EGFP were transfected into HeLa cells. As a positive control, we took along an EGFP fusion of the influenza A virus (IAV) NS1 protein, which is an established PKR antagonist. As shown in Fig 2E, plasmid DNA transfection induced SG formation except for the plasmids encoding the MERS-CoV p4a and IAV NS1 EGFP fusion proteins. The absence of SG formation (Fig 2E and 2F) coincided with a lack of PKR phosphorylation (Fig 2G). We also tested the ability of these MERS-CoV accessory proteins to suppress the stress response pathway induced by the more commonly applied method of poly(I:C) transfection. Again, we observed that p4a, but none of the other MERS-CoV accessory proteins, suppressed SG formation (S3 Fig). The inhibitory effect of p4a, as well as that of NS1, was less pronounced in this assay, possibly because the relatively large amounts of poly(I:C) may exceed the maximum capacity of the PKR antagonists. Taken together, our data suggests that MERS-CoV p4a is a PKR antagonist and inhibits the stress response pathway at the level of, or upstream of, PKR phosphorylation.

### MERS-CoV p4a suppresses PKR-mediated translation inhibition

**Fig 3:**
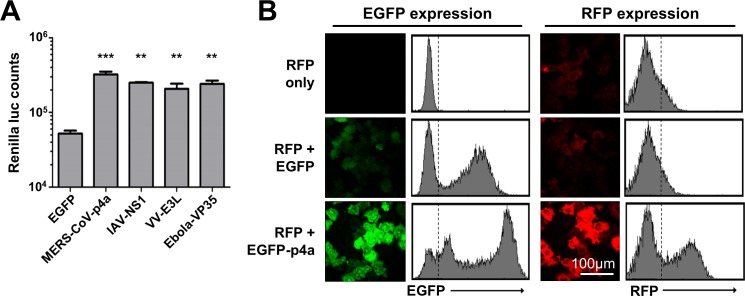
MERS-CoV p4a rescues protein translation upon plasmid DNA transfection-mediated stress. (A) Bar-graph showing Renilla luciferase counts measured at 16h post co-transfection of pTK-RLuc and pEGFP expression plasmids. Means and standard deviations are shown of triplicate measurements. Data was analyzed using an unpaired t-test (***, p<0.001; **, p<0.01). (B) Flow cytometry analysis of HeLa cells expressing RFP, RFP and EGFP, or RFP and EGFP-p4a. The dashed lines in the histograms divide non-RFP/EGFP expressing cells from RFP/EGFP-expressing cells.

We observed that the protein levels of p4a and NS1 were higher than those of the other MERS-CoV accessory proteins (Fig 2E). We reasoned that the inhibition of plasmid DNA-induced PKR activation may increase protein translation levels. Indeed, co-expression of p4a or NS1 together with *Renilla* luciferase (RLuc) caused a reproducible 5- to 10-fold increase in luciferase counts compared to the EGFP control plasmid (Fig 3A). This effect was attributed to increased translation, since p4a expression had no effect on RLuc mRNA levels. In addition, RLuc counts were not increased in PKR^KO^ cells, indicating that p4a increases translation efficiency via inhibition of PKR (S4 Fig). Other established viral PKR antagonists like the Vaccinia virus E3L [26] and Ebola virus VP35 [27] caused a similar increase in RLuc expression levels. Comparable results were obtained upon co-expression with an RFP expression plasmid (Fig 3B). These data are in line with the observation that MERS-CoV p4a antagonizes PKR activity, and provide another indication that viral PKR antagonists can rescue translation efficiency in cells in which the stress pathway is activated by (viral) dsRNA.

### MERS-CoV p4a fails to inhibit PKR-independent stress pathway activation

**Fig 4:**
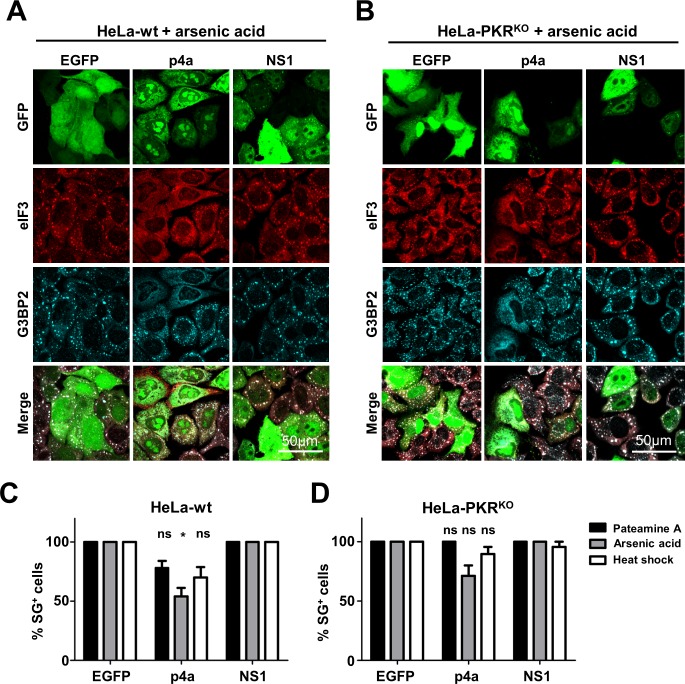
MERS-CoV p4a does not inhibit PKR-independent SG formation. (A, B) Immune fluorescence images of HeLa-wt cells (A) and HeLa-PKR^KO^ cells (B) transfected with the indicated pEGFP-expression plasmids. Next day, SG formation was triggered using arsenic acid (0.5 mM for 30 min). Cells were fixed and stained for eIF3 (shown in red) or G3BP2 (shown in cyan). EGFP expression is shown in green. (C, D) Quantification of SG-positive HeLa-wt cells (C) and HeLa-PKR^KO^ cells (D) treated with Pateamine A (100 nM for 2h), arsenic acid (0.5 mM for 30 min), or heat shock (50°C for 30 min). SG-positive cells were quantified from three randomly selected images. Shown are means with standard deviations, which were analyzed using an unpaired t-test. (*, p<0.05; ns, not significant).

Both MERS-CoV p4a and IAV NS1 are dsRNA binding proteins [6,21], which suggests that p4a shields the viral dsRNA from detection by PKR. To test whether p4a can also inhibit stress pathway activation via PKR- and dsRNA-independent mechanisms, we used arsenic acid and heat shock to induce eIF2α-dependent stress pathway activation [28]. Furthermore, we used pateamine A to induce SG formation via an eIF2α-independent mechanism [29]. In agreement with earlier findings, IAV NS1 failed to inhibit PKR-independent SG formation [30]. A small reduction in PKR-independent SG formation was observed in cells overexpressing p4a (Fig 4A and 4B). However, lack of SGs was only observed in cells expressing very high levels of p4a, whereas a moderate expression level of p4a was already sufficient to inhibit PKR-mediated SG formation (Fig 2E). To rule out any involvement of PKR expression in the small reduction of PKR-independent SG formation, we tested arsenic acid, heat shock and pateamine A-induced stress pathway activation in HeLa-PKR^KO^ cells. Also under these conditions, expression of p4a affected SG formation only in a small fraction of the cells (Fig 4C and 4D). Thus, MERS-CoV p4a seems to predominantly suppress dsRNA-dependent PKR activation and does not efficiently target other parts of the stress response pathway.

### MERS-CoV p4a can functionally replace the PKR antagonist of a picornavirus

Studying immune evasion functions of viral proteins by transient overexpression from plasmid DNA may suffer from shortcomings. Transfection procedures fail to mimic the dynamic interplay between dsRNA and the antagonist, both of which gradually appear over time during the viral life cycle. Furthermore, transfection may yield non-physiologically high levels of viral proteins and/or dsRNA mimics which may blur results. Also, dsRNA-mimicking molecules, like poly(I:C), may be delivered to compartments where viral dsRNA does not naturally localize under infection conditions.

Therefore, we set out to investigate the function of p4a as an innate antiviral response antagonist under infection conditions. For this, we made use of a recombinant encephalomyocarditis virus (EMCV, strain mengovirus). EMCV is a member of the picornavirus family that, like coronaviruses, produces dsRNA replication intermediates during its life cycle. In the recombinant EMCV, the function of the leader (L) protein–which antagonizes the dsRNA-triggered IFN-α/β and stress response pathways–is disturbed by specific mutations in an essential zinc-finger motif (EMCV-L-Zn) [31,32]. By consequence, and in contrast to wt virus, EMCV-L-Zn causes strong activation of the IFN-α/β and stress response pathways [31,32].

**Fig 5:**
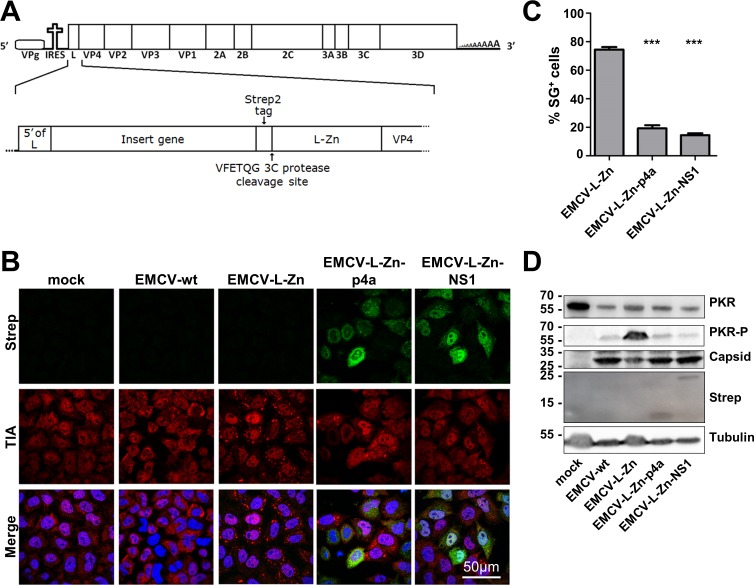
MERS-CoV p4a inhibits PKR activation during mengovirus infection. (A) Schematic overview of the recombinant mengovirus system. The upper panel shows the wt mengovirus genome. The lower panel highlights the 5’-region showing the gene insertion upstream of the inactivated L. (B) Immune fluorescence images of HeLa-wt cells that were mock-infected or infected with wt mengovirus or the indicated recombinant mengoviruses (MOI = 10). Cells were fixed at 6h post infection and stained for TIA1 (shown in red) and Strep-tagged p4a or NS1 (shown in green). Nuclei were stained using Hoechst-33258 (shown in blue). (C) SG-positive cells were quantified from three randomly selected images. Shown are means with standard deviations, which were analyzed using an unpaired t-test (***, p<0.001). (D) Western blot analysis of PKR and phospho-PKR in cells infected with indicated viruses. Capsid staining was used as a control for virus replication efficiency, tubulin staining was used as loading control and Strep-tag staining showed expression of the MERS-CoV p4a and IAV NS1.

To study whether heterologous expression of p4a can prevent PKR activation, recombinant viruses were generated expressing Strep2-tagged MERS-CoV p4a or IAV NS1 (as a control) upstream of the inactivated L (Fig 5A). EMCV wt infection did not induce SG formation while EMCV-L-Zn induced SGs in ~80% of the cells. Infection of cells with recombinant EMCV-L-Zn expressing p4a or NS1 protein resulted in SG formation in <20% of the cells (Fig 5B and 5C). This reduction was not due to differences in infection efficiency, since Strep2-tagged proteins were detected in the majority of cells (Fig 4B). In fact, SGs were only observed in cells displaying low expression levels of p4a or IAV NS1.

Western blot analysis was performed to assess the level of PKR phosphorylation. Total PKR levels were significantly reduced in EMCV-infected cells, a phenomenon that was described earlier by Dubois *et al*.[33], although the mechanism behind this remains unclear. Yet, even with these reduced PKR levels, EMCV-L-Zn infection induced strong PKR phosphorylation, which was reversed by the expression of p4a or NS1 (Fig 5D). Analysis of viral protein levels using an antibody directed against the viral capsid indicated that viral protein levels were higher in cells infected with p4a- and NS1-expressing viruses compared to EMCV-L-Zn infected cells, indicating that expression of these PKR antagonists increased virus replication efficiency. Taken together, these results indicate that MERS-CoV p4a can functionally replace the PKR antagonist of a picornavirus in infected cells.

### The MERS-CoV p4a dsRNA-binding domain is crucial for its function

**Fig 6:**
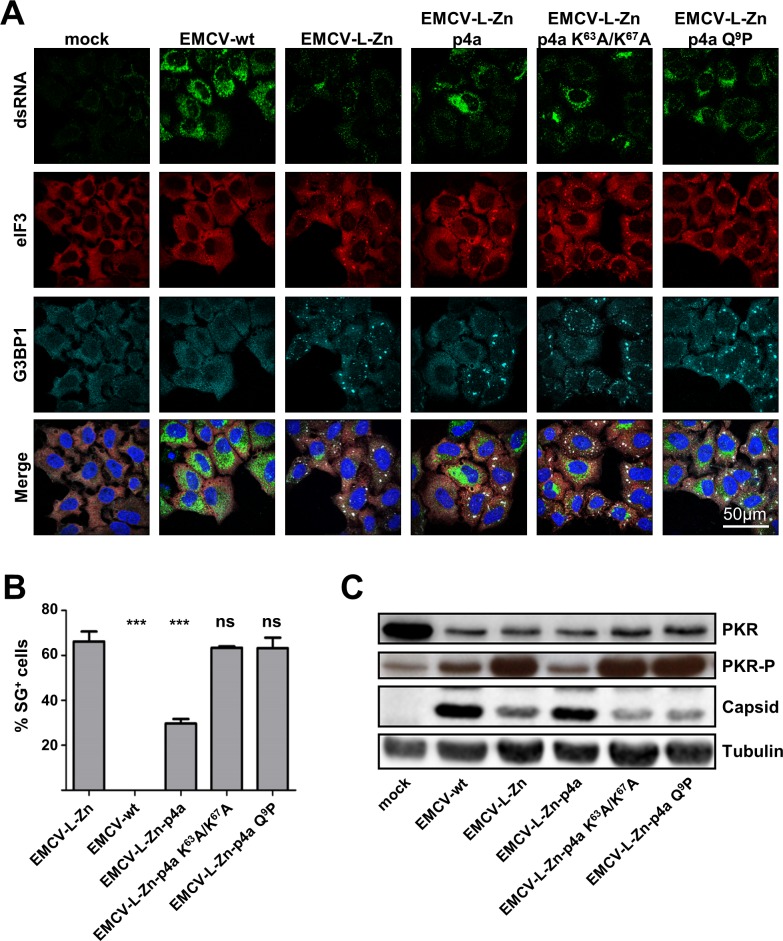
The dsRNA binding motif in MERS-CoV p4a is crucial for suppressing SG formation. (A) Immune fluorescence images of HeLa-wt cells that were mock-treated or infected with wt mengovirus or the indicated recombinant mengoviruses (MOI = 10). Cells were fixed at 6h post infection and stained for dsRNA (shown in green), eIF3 (shown in red), and G3BP1 (shown in cyan). Nuclei were stained using Hoechst-33258 (shown in blue). (B) SG-positive cells were quantified from three randomly selected images. Shown are means with standard deviations, analyzed using an unpaired t-test (***, p<0.001; ns, not significant). (C) Western blot analysis of PKR and phospho-PKR in cells infected with indicated viruses. Capsid staining was used as a control for virus replication efficiency and tubulin staining was used as loading control.

MERS-CoV p4a contains a dsRNA-binding motif similar to those found in some cellular proteins (S5 Fig). Previously, a p4a mutant containing substitutions in its dsRNA-binding motif (K^63^A/K^67^A) was shown to be deficient in binding dsRNA [22]. Based on the sequence similarity of this dsRNA-binding motif to those in Staufen, ADAR1, ADAR2 and PKR, and the published NMR structure of the ADAR2 dsRNA-binding domain in complex with its ligand [34], we designed a second mutant containing a single substitution (Q^9^P) in another part of the conserved dsRNA-binding motif (S5 Fig). Infection of HeLa cells with recombinant EMCV-L-Zn viruses expressing either of these p4a mutants resulted in efficient SG formation, indicating a complete loss of the stress-antagonizing function (Fig 6A and 6B). In agreement herewith, analysis of the PKR phosphorylation status demonstrated that the p4a mutants failed to inhibit PKR phosphorylation (Fig 6C). Consistently, viruses expressing these mutants showed reduced capsid protein expression, possibly as a consequence of PKR-mediated translation inhibition. Thus, the dsRNA-binding motif in MERS-CoV p4a is essential for its function to antagonize PKR-mediated SG formation and translation shut-off.

### Expression of MERS-CoV p4a also suppresses IFN-α/β pathway activation under infection conditions

**Fig 7:**
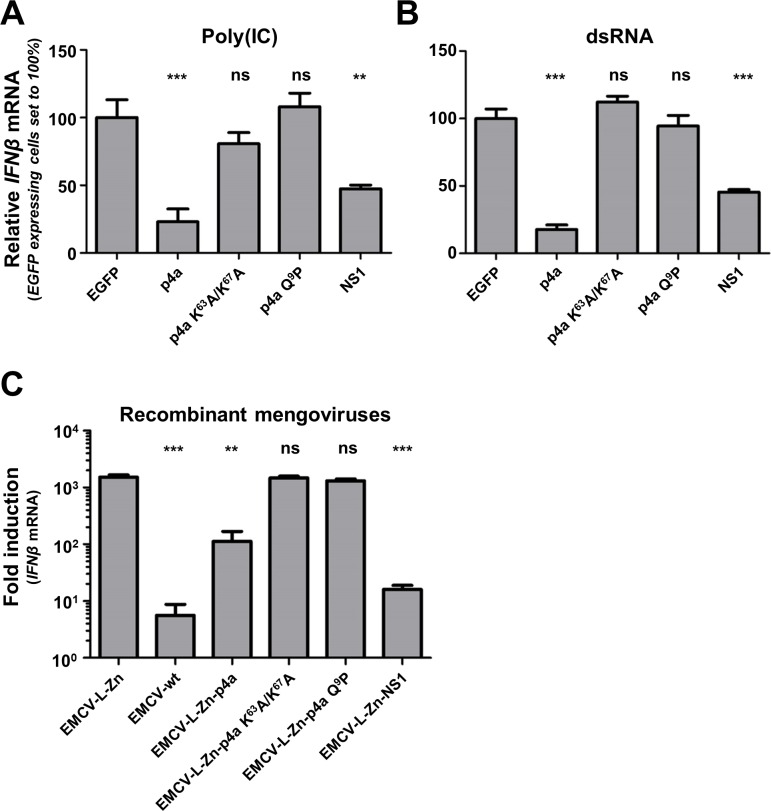
MERS-CoV p4a is a type I IFN antagonist. (A, B) Relative *IFNβ* mRNA levels induced by transfection of poly(I:C) (A) or 6.5 kb viral dsRNA (sequence derived from the Coxsackie virus B3 genome) (B) in HeLa-wt cells expressing EGFP or EGFP-p4a fusion proteins. To obtain a cell pool in which all cells express the protein of interest, plasmids encoding EGFP fusion proteins were co-transfected with a plasmid conferring puromycin resistance. Subsequent puromycin selection for two days eliminated non-transfected cells. RT-qPCR was used to quantify relative *IFNβ* mRNA levels 8h post RNA ligand transfection. Shown are means and standard deviations of the relative *IFNβ* mRNA levels compared to EGFP-expressing cells. Analysis was performed by unpaired t-test (***, p<0.001; **, p<0.01; ns, not significant). (C) Bar-graph showing *IFNβ* mRNA levels induced by recombinant mengovirus infection (MOI = 10) of HeLa cells. RT-qPCR was used to quantify relative *IFNβ* mRNA levels at 8h post infection. Means and standard deviations of the relative *IFNβ* mRNA levels of triplicates are shown and analyzed using an unpaired t-test (***, p<0.001; **, p<0.01; ns, not significant).

Previous studies have shown that expression of p4a is able to reduce the level of IFN-α/β pathway activation in transiently transfected cells [21–23]. Consistently, we observed that transient expression of p4a inhibited poly(I:C)-induced (Fig 7A) and dsRNA-induced (Fig 7B) *IFNβ* mRNA transcription. To assess whether p4a can also inhibit the IFN-α/β pathway in virus-infected cells, we compared *IFNβ* mRNA transcription levels in cells infected with recombinant EMCV-L-Zn viruses expressing either p4a or NS1. Both p4a and IAV NS1 significantly suppressed transcription of *IFNβ* mRNA (Fig 7C). This ability was lost in viruses expressing mutant p4a proteins that are unable to bind dsRNA (Fig 7). These data show that MERS-CoV p4a also inhibits the IFN-α/β response in EMCV-L-Zn-infected cells and that this function also requires its dsRNA-binding activity.

### MERS-CoV p4a increases EMCV-L-Zn replication efficiency

**Fig 8:**
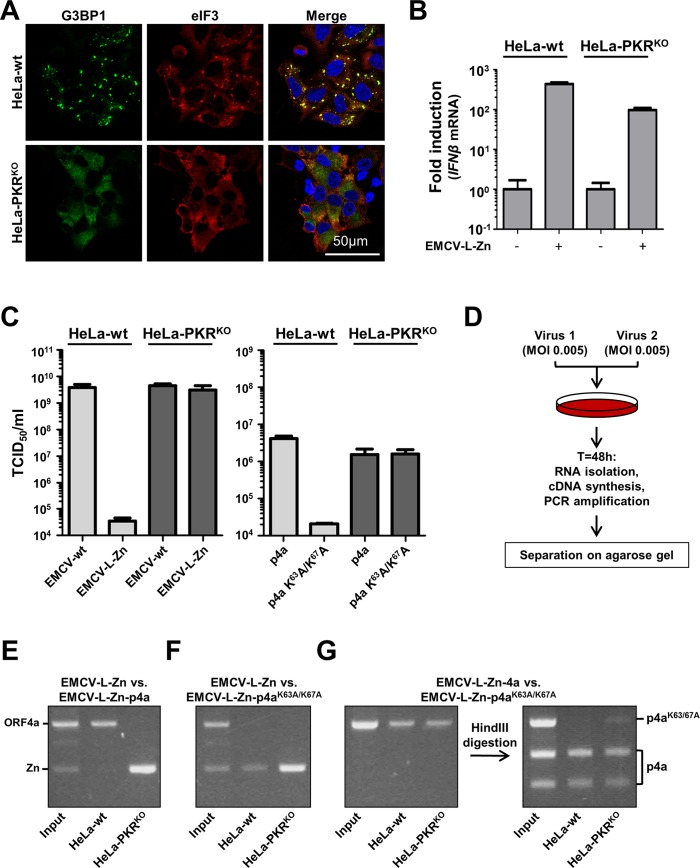
MERS-CoV p4a increases mengovirus fitness. (A) Immune fluorescence images of HeLa-wt and HeLa-PKR^KO^ cells infected with EMCV-L-Zn (MOI = 10). Cells were fixed at 6h post infection and SG formation was visualized using antibodies directed against G3BP1 (shown in green) and eIF3 (shown in red). Nuclei were stained using Hoechst-33258 (shown in blue). (B) In parallel with *A*, RNA was isolated at 8h post infection and relative *IFNβ* mRNA levels were quantified by RT-PCR. Means and standard deviations of triplicate measurements are shown. (C) Virus production after wt and recombinant mengovirus infection (MOI = 0.01) in HeLa and HeLa-PKR^KO^ cells. Supernatant was collected 24h post infection and virus progeny was titrated by end-point dilution with 3-fold dilution steps. (D) Schematic representation of the virus competition assay. Briefly, two viruses are mixed 1:1 and used to infection HeLa-wt or HeLa-PKR^KO^ cells. Progeny virus was collected 48h post infection and viral RNA was isolated. RT-PCR was used to amplify the MERS-CoV 4a insert, which was analyzed using agarose gel electrophoresis. (E, F, G) Agarose gel analysis of the 4a insert region from virus competition assays with the indicated viruses. To distinguish between wild-type and mutant 4a genes, 4a-wt specific HindIII digestion was used.

Our data show that p4a is a multi-functional protein that antagonizes both the stress response and the IFN-α/β response pathways. To demonstrate the functional and beneficial role of p4a-mediated antagonism of the stress response pathway, we set out to compare the replication efficiency of recombinant viruses in HeLa-wt cells and cells that are defective in the PKR-induced stress response pathway (HeLa-PKR^KO^ cells). Infection of HeLa-PKR^KO^ cells with EMCV-L-Zn showed that these cells are unable to mount a stress response (Fig 8A), whereas IFN-α/β pathway activation was only slightly affected in these cells (Fig 8B), indicating that possible differences in virus fitness can be predominantly attributed to the defective stress response pathway. Replication of EMCV-L-Zn under low MOI infection conditions is severely impaired in HeLa-wt cells, whereas replication was fully rescued to the level of EMCV wt in HeLa-PKR^KO^ cells (Fig 8C). Comparison of the replication efficiency of recombinant viruses expressing p4a or the p4a mutant containing the K^63^A/K^67^A substitutions showed that the antagonistic activity of p4a provided a clear fitness advantage in HeLa-wt cells (Fig 8C). The observation that the p4a-expressing virus failed to replicate to similar titers as wt virus is unlikely due to inefficient PKR inhibition by p4a as comparable titers were obtained for the recombinant viruses expressing p4a or mutant p4a in HeLa-PKR^KO^ cells. Notwithstanding the lower virus titer, which may either be due to imperfect polyprotein processing due to introduction of p4a or to less efficient encapsidation of the larger viral genome, these results provide evidence that the PKR antagonistic function of MERS-CoV p4a can provide a virus fitness advantage in PKR-competent cells.

Similar results were obtained in virus competition experiments (Fig 8D), which is a more sensitive method to compare virus fitness and can reveal smaller fitness differences. Upon low MOI infection, EMCV-L-Zn expressing p4a rapidly outgrew EMCV-L-Zn in HeLa-wt cells but not in HeLa-PKR^KO^ cells (Fig 8E). No fitness advantage was observed with virus expressing the mutant p4a (Fig 8F). We also co-infected cells with viruses expressing either p4a or mutant p4a. Since these viruses could not be distinguished based on their amplicon length, we used a HindIII restriction reaction to specifically cleave the wt 4a PCR fragment (the HindIII site is absent in the mutant 4a gene). Consistent with the results of the multi-cycle infection experiment shown in Fig 8C, the virus expressing p4a replicated better than the virus expressing mutant p4a in HeLa-wt cells whereas in PKR^KO^ cells only a minor advantage was observed (Fig 8G).

### MERS-CoV encodes at least one other suppressor of the stress response pathway

**Fig 9:**
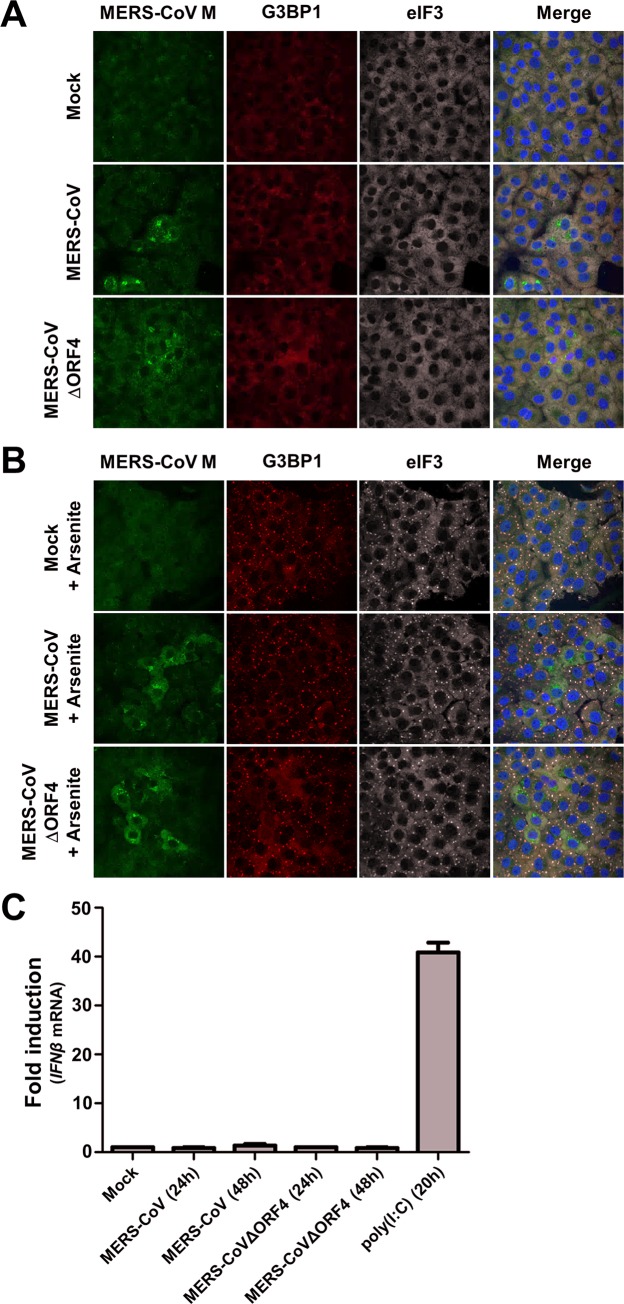
MERS-CoV encodes another suppressor of innate antiviral responses. (A, B) Vero cells were infected (MOI = 1) with MERS-CoV wt or MERS-CoVΔORF4. At 16h p.i., cells were (A) mock treated, or (B) treated with 0.5 mM arsenic acid for 1h. Subsequently, MERS-CoV infection and SG formation were visualized by IFA using antibodies directed against MERS-CoV M, G3BP1, and eIF3, respectively. (C) Huh7 cells were transfected with poly(I:C), or infected (MOI = 1) with the indicated viruses. RT-qPCR was used to quantify relative *IFNβ* mRNA levels at the indicated time points. Shown are means and standard deviations of the relative *IFNβ* mRNA levels compared to mock treated cells.

Thus far, we used a recombinant picornavirus, EMCV, to analyze the function of p4a in virus-infected cells, in the absence of other MERS-CoV proteins. To assess the relevance of p4a for stress response antagonism in MERS-CoV infected cells, we used recombinant MERS-CoVΔORF4 that is deficient in p4a and p4b expression. Surprisingly, like wt MERS-CoV, MERS-CoVΔORF4 did not induce SG formation in Vero cells (Fig 9A), suggesting that MERS-CoV expresses at least one other protein that suppresses the stress response pathway. To gain more insight into the working mechanism of this other stress response pathway antagonist, we treated MERS-CoV infected cells with arsenite. As demonstrated in Fig 9B, this treatment resulted in SG formation in all the uninfected cells, whereas no SGs were detected in cells infected with either MERS-CoV or MERS-CoVΔORF4 (Fig 9B). These findings strongly suggest that MERS-CoV encodes at least one other stress response antagonist with a mode of action that differs from that of p4a. We also tested the IFN-α/β pathway activation in cells infected with the mutant virus. In line with the reports that several MERS-CoV proteins can antagonize the IFN-α/β pathway [21,23,35–38], no increase in *IFNβ* mRNA levels was observed in Huh7 cells infected with MERS-CoV or MERS-CoVΔORF4 (Fig 9C). Taken together, these data provide evidence for substantial redundancy with respect to antagonism of innate antiviral responses in MERS-CoV infected cells.

## Discussion

Most viruses have evolved mechanisms to antagonize innate antiviral responses. Coronaviruses encode a set of genus-specific, or in some cases even species-specific, proteins that are generally dispensable for replication *in vitro* but ensure efficient virus replication and/or spreading *in vivo* [10,11,39–41]. Some of these so-called accessory proteins have been shown to antagonize specific innate antiviral responses, but the functions of most of them are still unknown [9,10,23,42–44]. Thus far, most studies concentrated on IFN-α/β pathway antagonists, whereas inhibition of the cellular stress response pathway by coronaviruses remains largely unexplored. In this study, we focused on the recently identified MERS-CoV, and showed that infected cells fail to activate the stress response pathway. In our subsequent search for MERS-CoV-encoded stress response antagonists, each of its accessory proteins was tested individually for the ability to suppress this pathway. Transient expression of p4a specifically suppressed dsRNA-mediated and PKR-dependent translation inhibition and SG formation. Moreover, we showed that p4a can functionally substitute for the PKR antagonist of EMCV in infected cells. Introduction of specific mutations revealed that the ability of p4a to suppress activation of the stress response pathway depends on its dsRNA-binding function. Together, the data strongly suggest that p4a suppresses the PKR-mediated stress response pathway by sequestering viral dsRNA. Yet, infection of cells with a recombinant MERS-CoV deficient in p4a expression failed to trigger SG formation. This finding points to the expression of at least one other stress response antagonist by MERS-CoV. Importantly, this other suppressor(s) differs in its mode of action of p4a, since in contrast to p4a, it was able to suppress activation of the arsenite-induced stress pathway. Together, these data suggest that MERS-CoV has evolved redundant mechanisms to suppress the stress response pathway at multiple levels.

To our knowledge, MERS-CoV p4a is the first coronavirus protein identified as an antagonist of the dsRNA-dependent, PKR-mediated stress response. There are strong indications that other coronaviruses also encode stress response antagonists but their identity and mode of action remain to be determined. Infectious bronchitis virus (IBV, a γ-CoV) interferes with phosphorylation of both PKR and eIF2α through an unknown mechanism(s) [45]. Transmissible gastroenteritis virus (TGEV) Transmissible gastroenteritis virus (TGEV) triggers SG formation, but causes a reduction in the amount of phosphorylated eIF2α over time, possibly by recruiting eIF2α phosphatase PP1 through accessory protein 7 [46,47]. Mouse hepatitis virus (MHV, a lineage A β-CoV) triggers eIF2α phosphorylation and SG formation relatively late in infection, suggesting that the virus actively delays the stress response pathway [48–50], but the mechanism is unknown. SARS-CoV (a lineage B β-CoV) has been reported to trigger PKR activation but to be resistant to its antiviral activity [51], although in another study a strong antiviral effect of PKR was observed [52]. Hence, the limited information that is available suggests that coronaviruses have acquired different strategies to antagonize the stress response pathway. Importantly, none of these coronaviruses encode a protein with any homology to MERS-CoV p4a.

In this study, we assessed p4a’s antagonistic activities not only upon transient overexpression, but also in the context of viral infection. For this, we introduced p4a in a recombinant EMCV (EMCV-L-Zn) in which the IFN-α/β and stress response pathway antagonist—the leader (L) protein—was inactivated. A p4a-expressing recombinant EMCV may provide several advantages over overexpression through transient transfection, as it likely better mimics the dynamic production of—as well as the interplay between—dsRNA and the viral antagonist. Using this approach, we showed that dsRNA sequestration by p4a efficiently suppresses the PKR-dependent stress response pathway as well as MDA5-mediated IFN-α/β responses under these infection conditions, and thereby provides a fitness advantage to this recombinant EMCV. Similar results were obtained with a recombinant virus expressing IAV NS1, which was included as a control. Together, these data suggests that p4a can be categorized in the group of previously identified viral dsRNA-binding antagonists of stress response and IFN-α/β pathways, which besides IAV NS1, also includes Ebola virus VP35 and Vaccinia virus E3L [26,27].

Our results showed that besides p4a, MERS-CoV expresses at least one other stress response antagonist. This other antagonist(s) is likely one of the nsps or a structural protein, as we excluded stress-antagonizing roles of the other accessory proteins. At least one antagonist can also suppress the arsenite-induced stress response pathway, and is therefore unlikely to act directly at the level of PKR. Instead, it may act at the level of eIF2α phosphorylation or SG formation. Identification of the other stress response antagonist(s) and elucidation of its/their mode of action, awaits further investigation.

Functional redundancy in suppressing innate antiviral responses is a well-documented phenomenon for coronaviruses. The MERS-CoV accessory proteins (p4a, p4b, and p5) [21–23,36] as well as the structural M protein and the ORF1ab-encoded nsp3 [38,37], have all been implicated in antagonizing IFN-α/β pathway activation. This provides a likely explanation for our observation that recombinant MERS-CoV lacking p4a and p4b was still able to suppress *IFN-β* mRNA transcription. MERS-CoV p4a homologs have exclusively been identified in lineage C β-CoVs, which besides MERS-CoV comprises a MERS-like coronavirus found in European hedgehogs [53], and bat coronaviruses (BatCoV) HKU4 and HKU5 [54–56]. The p4a-like accessory proteins of these other lineage C viruses all contain dsRNA-binding motifs and may therefore have similar functions as MERS-CoV p4a. Yet, a study by Siu *et al*. indicated that p4a of BatCoV-HKU4, in contrast to that of MERS-CoV and BatCoV-HKU5, does not bind poly(I:C) and does not inhibit IFN-α/β responses [22]. If BatCoV-HKU4 p4a is indeed unable to sequester dsRNA, then it is likely unable to suppress the dsRNA-triggered stress response pathway as well. Interestingly, sequence analysis of a MERS-CoV strain isolated from patients in Jordan identified a 16 amino acid deletion in p4a [57]. This deletion does not affect the residues comprising the dsRNA binding site. However, as it removes the second β-strand in the classical αβββα-fold of the dsRNA binding domain, p4a’s dsRNA binding properties and, in consequence, its function as antagonist, are most likely compromised. If so, stress antagonism by p4a may be dispensable for MERS-CoV replication and transmission among humans. Increasing evidence suggests that coronavirus accessory proteins often have niche-specific (e.g. organ- or tissue-specific) or host-tailored functions. For example, accessory protein 3c is required for replication of low-virulence feline enteric coronavirus (FECV), which primarily replicates in the enteric tract, but not for replication of FECV-derived, highly virulent feline infectious peritonitis virus (FIPV) isolates, which have acquired the ability to replicate in macrophages [58,59]. Also, accessory proteins contributing to viral fitness in one particular host species may sometimes prove less important in a novel host following a species-jump. For example, in SARS-CoV and CoV-229E some accessory genes were lost through gradual deletion following the introduction of these viruses into humans [60,61]. Acquisition as well as loss of accessory proteins may reflect adaptations to different immunological environments in different niches or hosts. In this study, we showed that MERS-CoV p4a can potently antagonize innate antiviral responses in human cells. Yet, as suggested by the Jordan outbreak, p4a may not be critical for zoonotic transmission nor for limited human-to-human spread, possibly because of redundancy in viral anti-stress response strategies. Whether p4a will be lost or maintained in the hapless event MERS-CoV establishes sustained community transmission remains an open question.

## Materials and Methods

### Cell culture and viruses

HeLa-R19, Huh7 and BHK-21 cells were maintained in Dulbecco’s Modified Eagle’s Medium (DMEM) supplemented with 10% (V/V) fetal calf serum (FCS). Vero cells (ATCC CCL-81) were grown in Eagle’s minimum essential medium with 8% FCS, 100 units/ml penicillin and streptomycin, 2 mM L-glutamine and non-essential amino acids.

MERS-CoV infections [62] were carried out as described previously [24,63] inside biosafety cabinets in BSL III facilities at Leiden University Medical Center and Universidad Autonoma de Madrid. Recombinant MERS-CoVs that were used in Madrid have been described previously [63]. Recombinant MERS-CoVs that were used in Leiden were derived from the previously described infectious MERS-CoV clone pBAC-MERS^FL^ [63], and adapted as follows using two step en-passant *in vivo* recombineering reactions in *E*. *coli* [64]. The CMV promoter at the 5’end of the MERS-CoV cDNA sequence was replaced by a T7 RNA polymerase promoter and a unique NotI linearization site was inserted at the 3’end, so that the virus could be launched from transfecting *in vitro* synthesized RNA transcripts (produced using an mMESSAGE mMACHINE T7 transcription kit from ThermoFisher scientific). To construct MERS-CoVΔORF4 from this adapted clone, the coding sequence of MERS-CoV p4a/p4b was removed and replaced by a red fluorescent protein (RFP) gene, which however for unclear reasons did not result in red fluorescence during infection. All the genetic modifications to the original pBAC-MERS^FL^ were verified by sequencing. The MERS-CoVΔORF4 virus grew to similar titers as the recombinant wt MERS-CoV derived from the original clone.

Recombinant EMCV viruses were derived from the pM16.1 infectious clone [65]. The pStrep2-VFETQG-Zn-M16.1 infectious clone was constructed using site-directed mutagenesis (SDM) using the pCVB3-3C^pro^-QG-M16.1 as template DNA [32]. The Zn-finger mutation in L was introduced by SDM using the following oligonucleotides: Fw; 5’-ATGACCTTTGAAGAAGCCCCAAAAGCCTCCGCCTTACAATAC-3’ and Rv; 5’- GGAATGAGCACAAATCTCTTG-3’. The optimized 3C^pro^ recognition site (VFETQG) was introduced by SDM using the following oligonucleotides: Fw; 5’-GAAACTCAAGGCGCAACGACTATGGAGC-3’ and Rv; 5’-AAAGACCGCGGCCGCTTGCTCATCATTG-3’. Finally, the Strep2-tag was introduced by SDM using the following oligonucleotides: Fw; 5’-GGCCGCCTGGTCACATCCTCAGTTTGAGAAGGGTGCCTGGTCTCATCCCCAATTCGAAAA-3’ and Rv: 5’- GGCCTTTTCGAATTGGGGATGAGACCAGGCACCCTTCTCAAACTGAGGATGTGACCAGGC-3’. Genes of interest were inserted into the XhoI/NotI restriction sites of the pStrep2-VFETQG-Zn-M16.1 infectious clone. Viruses were recovered by transfection of run-off RNA transcripts into BHK-21 cells. Upon total CPE, cells were subjected to three freeze-thaw cycles and cell debris was pellet at 4,000x*g* for 15 minutes. Virus was concentrated by ultracentrifugation though a 30% sucrose cushion at 140,000x*g* for 16 hours in a SW32Ti rotor.

### HeLa-PKR knockout cells

HeLa-R19 PKR^KO^ were generated using the CRISPR/Cas9 system as previously described [66]. Briefly, gRNA encoding oligonucleotides cassettes to target human PKR (gRNA1: 5’-ACCGGACCTCCACATGATAGG-3’ and 5’-AACCCTATCATGTGGAGGTCC-3’, gRNA2: 5’-CCGTACTACTCCCTGCTTCTGAG-3’ and 5’-AAACTCAGAAGCAGGGAGTAGTA-3’) were cloned into the SapI restriction sites of the pCRISPR-hCas9-2xgRNA-Puro plasmid. HeLa-R19 cells were seeded in 6-well clusters (100,000 cells/well) and next day transfected with 2.5 μg plasmid DNA using Fugene6 (Promega) according to manufacturer’s instructions. Next day successfully transfected cells were selected using puromycin and single-cell clones were generated using end-point dilutions. Knockout efficiency was determined by sequence analysis of the PKR locus in the genomic DNA and western blot analysis (S1 Fig).

### Chemicals and RNA ligands

Arsenic acid was purchased at Sigma-Aldrich and used at a final concentration of 0.5 mM in DMEM. Pateamine A was kindly provided by Prof. Jerry Pelletier [67] and used at a concentration of 100 nM in DMEM. Poly(I:C) was purchased from GE Healthcare and dsRNA ligand was prepared using the Replicator RNAi kit (Finnzymes) using the following oligonucleotides (Fw, possessing T7 promoter sequence) TAATACGACTCACTATAGGGGATACAGTGACAGGGCG and (Rv, possessing Phi6 promoter sequence) GGAAAAAAACCGCACCGAATGCGGAGAATTTAC and the pRib-CVB3/T7 Coxsackie virus B3 infectious clone as template [68].

### Plasmids

Expression plasmids encoding enhanced green fluorescent protein (EGFP) tagged proteins were created by PCR amplification of the gene of interest with oligonucleotides flanked by XhoI (Fw) or BamHI (Rv) restriction sites (MERS-CoV ORF3: 5’-AAAAACTCGAGATGAGAGTTCAAAGACCACCC-3’ and 5’-AAAAAGGATCCATTAACTGAGTAACCAACGTCAAAAAG-3’, ORF4a: 5’-AAAAACTCGAGATG GATTACGTGTCTCTGCTTAATC-3’ and 5’-AAAAAGGATCCGTGGGAGAATGGCTCCTC-3’, ORF4b: 5’-AAAAACTCGAGATGGAGGAATCCCTGATGGATG-3’ and 5’-AAAAAGGATCCAAA TCCTGGATGATGTAAAATGGGG-3’, ORF5: 5’-AAAAACTCGAGATGGCTTTCTCGGCGTC-3’ and 5’-AAAAAGGATCCAACGATAAGCGAGCTCGG-3’, IAV NS1: 5’-AAAAACTCGAGATGGAT CCAAACACTGTGTC-3’ and 5’-AAAAAGGATCCAACTTCTGACCTAATTGTTC-3’, VV E3L: 5’-AAAAACTCGAGATGTCTAAGATCTATATTGACGAGCGTTCTG-3’ and 5’-AAAAAGGATCCG AATCTAATGATGACGTAACCAAGAAGTTTATCTACTG-3’, Ebola VP35: 5’-AAAAACTCGAGATGAC AACTAGAACAAAGGGCAGGG-3’ and 5’-AAAAAGGATCCAATTTTGAGTCCAAGTGTTTTACC ATCTTGAAGC-3’. Digested PCR products were ligates into XhoI/BamHI digested pEGFP-N3 plasmid and gene integrity was confirmed by sequencing analysis. pcDNA-RFP expression plasmid was constructed by PCR amplification of the RFP gene using oligonucleotides flanked by NheI (Fw) and NotI (Rv) restriction sites (Fw) GCTAGCGCCACAACCATGGCCTCCTCCGAGGAC and (Rv) GCGGCCGCCGGCGCCGGTGGAGTGGCGGCCCTC and subsequently cloning into the NheI/NotI digested pcDNA-EGFP plasmid [69]. The pJET-puro (puromycin resistance vector) plasmid was developed by ligation of the EF1a-Puro fragment into the pJet1.2/blunt vector (Thermo Fisher). pRL-TK (Renilla luc expression vector) plasmid was purchased from Promega.

### Renilla luciferase assay

HeLa-R19 cells were seeded in a 96-wells cluster (5,000 cells/well) and the next day they were transfected with the indicated plasmids (40 ng pEGFP, 10 ng pRL-TK) using Fugene6. 24 hours post transfection, cells were lysed in 20 μl passive lysis buffer (Promega) and analyzed on the Centro LB 960 Microplate Luminometer (Berthold technologies) using the Renilla luciferase reporter kit (Promega) according to manufacturer instructions.

### Flow cytometry analysis

Cells were seeded in a 24-wells cluster (50,000 cells/well) and the next day they were transfected with the indicated plasmids (500 ng/well; 250 ng/plasmid) using Fugene6. Twenty-four hours post transfection, cells were released using trypsin, washed once in phosphate buffered saline (PBS) and fixed for 30 minutes with 2% paraformaldehyde (PFA) in PBS. Cells were analyzed on FACS Canto (BD) using BD FACS Diva software.

### Immunofluorescence assay (IFA)

Cells were seeded on glass slides in a 24 wells cluster (25,000 cells/well) and the next day they were infected (MOI = 10) or transfected (500 ng total DNA) using Fugene6. At 6h post infection or 24h post transfection, cells were fixed using 4% PFA in PBS for 30 minutes at RT. Vero cells seeded on glass slides were transfected with 1 μg Poly(I:C) per 6-well using Lipofectamine 2000 (Thermo Fisher Scientific). Cells were permeabilized with PBS + 0.2% Triton X-100, washed trice with blocking buffer (PBS + 2% bovine serum albumin [BSA] + 50mM NH_4_Cl), and incubated with blocking buffer for 1 h. Cell monolayers were incubated for 1 h with primary antibody mouse-α-G3BP1 (BD, 1:1,000), rabbit-α-TIA1 (Santa-Cruz, 1:50), mouse-α-dsRNA (J2, English&Scientific Consulting, 1:1,000), goat-α-eIF3 (Santa-Cruz, 1:100), rabbit-α-G3BP2 (Bethyl Laboratory, 1:200; or Assay Biotech, 1:500), or rabbit-α-MERS-CoV (1:500: raised against the MERS-CoV M carboxyl terminal peptide CRYKAGNYRSPPITADIELALLRA), and then for 30 min with secondary antibody donkey-α-mouse-Cy3 (Jackson ImmunoResearch, 1:1000), donkey-α-rabbit-Alexa488 (Jackson ImmunoResearch, 1:1000), bovine-α-goat-Alexa647 (Jackson ImmunoResearch, 1:1000), donkey-α-rabbit-Cy5 (Jackson ImmunoResearch, 1:200), donkey-α-mouse-Alexa 488 (Invitrogen, 1:200) or donkey-α-goat-Alexa 594 (Invitrogen, 1:200) and Hoechst-33258 (1:2,000) diluted in blocking buffer. Between and after the incubations, the cell monolayers were washed three times with blocking buffer. Finally, the cells were washed once with distilled water and coverslips were mounted on glass slides in FluorSafe (Calbiochem). Cells were examined by confocal microscopy (Leica SPE-II).

### Western blot analysis

Cells were seeded in 10-cm dishes (2.5 x 10^6^ cells/dish) and the next day cells were infected (MOI = 10) or transfected (8 μg plasmid DNA) using Fugene6. At 6h post infection or 24h post transfection, cells were released using trypsin, washed once in wash buffer (100 mM Tris/HCl pH 8,0 + 1 mM EDTA + 50 mM NaCl) and lysed in 200 μl lysis buffer (100 mM Tris/HCl pH 8,0 + 1 mM EDTA + 50 mM NaCl + 1% NP40 + protease inhibitor mix [Roche] + phosphatase inhibitor cocktails #2 and #3 [Sigma-Aldrich]). Cell debris was pelleted at 15,000 x *g* for 15 min and 10 μl of cleared cell lysates were resolved using reducing sodium dodecyl sulfate-polyacrylamide gel electrophoresis (SDS-PAGE) and transferred to 0.2 μm nitrocellulose membranes by wet electrophoretic transfer. Membranes were washed once with washing buffer (PBS + 0.1% Tween 20) and incubated 1h in blocking buffer (PBS + 0.1% Tween 20 + 2% BSA). Membranes were successively incubated for 1 h with primary antibody mouse-α-PKR (BD, 1:1,000), rabbit-α-PKR-P[T446] (Abcam, 1:2.000), mouse-α-Tubulin (Sigma, 1:5.000), rabbit-α-mengovirus capsid (kindly provided by Prof. Ann Palmenberg, 1:1.000) or mouse-α-StrepMab classic (IBA, 1:1.000) and then for 30 min with goat-α-mouse-IRDye680 (Li-COR, 1:15,000) or goat-α-rabbit-IRDye800 (Li-COR, 1:15,000) diluted in blocking buffer. Between and after the incubations, the membranes were washed, thrice each time, with washing buffer. Finally, membranes were washed once with PBS and scanned using an Odyssey Imager (Li-COR).

### RT-qPCR analysis

RNA isolation, cDNA synthesis, and RT-qPCR were performed as described elsewhere [66,63].

## Supporting Information

S1 Fig Construction of PKR knockout HeLa cells using the CRISPR-Cas9 system. (A) Schematic representation of the PKR gene. Two guide RNAs were designed to target exon 3 of human PKR. (B) A single-cell clone was characterized by isolation of genomic DNA and integrity of human PKR gene was determined by sequence analysis. Both alleles contain a deletion resulting in a frame-shift event and a premature stop codon. (C) Western blot analysis of PKR protein levels in cell lysates from HeLa-wt or HeLa-PKR^KO^ cells.
(TIF)

S2 Fig Stress granule formation in HeLa cells transfected with different plasmids. HeLa cells were transfected with different plasmids (500 ng/well). At 24h post transfection, cells were fixed and IFA was used to quantify the level of cells that possess SGs. For each type of plasmid, SG-positive cells were quantified from three randomly selected images and depicted in a bar-graph.
(TIF)

S3 Fig MERS-CoV p4a suppresses poly(I:C)-induced SG formation. HeLa-wt cells were transfected with pEGFP-expression plasmids. Next day, SG formation was triggered by poly(I:C) transfection (100 ng/well). Cells were fixed using paraformaldehyde at 6h post RNA ligand transfection and SG formation was visualized using IFA. Quantification of SG-positive cells is shown as means and standard deviations of at least three randomly selected images per sample. Data was analyzed using an unpaired t-test (***, p<0.001; *, p<0.05; ns, not significant).
(TIF)

S4 Fig Increased transgene expression is caused by a rescue of translation efficiency. (A, B) Relative *luciferase* mRNA (A) and protein (B) levels in HeLa-wt cells co-transfected with pTK-RLuc and pEGFP expression plasmids. (C) Relative luciferase counts measured at 16 h post co-transfection of pTK-RLuc and pEGFP expression plasmids in Hela-PKR^KO^ (D) cells. Data was analyzed using an unpaired t-test (***, p<0.001; *, p<0.05; ns, not significant).
(TIF)

S5 Fig The MERS-CoV p4a dsRNA binding motif. (A) Alignment of MERS-CoV p4a with other dsRNA binding motifs of several cellular proteins. In bold are the conserved residues crucial for dsRNA binding. (B) Structure of ADAR1 dsRNA binding motif in association with dsRNA. Highlighted are the corresponding ADAR1 residues that are mutated in MERS-CoV p4a in this study.
(TIF)
