# Supplementary figures and images for: Middle East Respiratory Coronavirus Accessory Protein 4a Inhibits PKR-Mediated Antiviral Stress Responses
Source: PLoS Pathog. 2016 Oct 26;12(10):e1005982. doi: 10.1371/journal.ppat.1005982 (PMC5081173; doi:10.1371/journal.ppat.1005982)

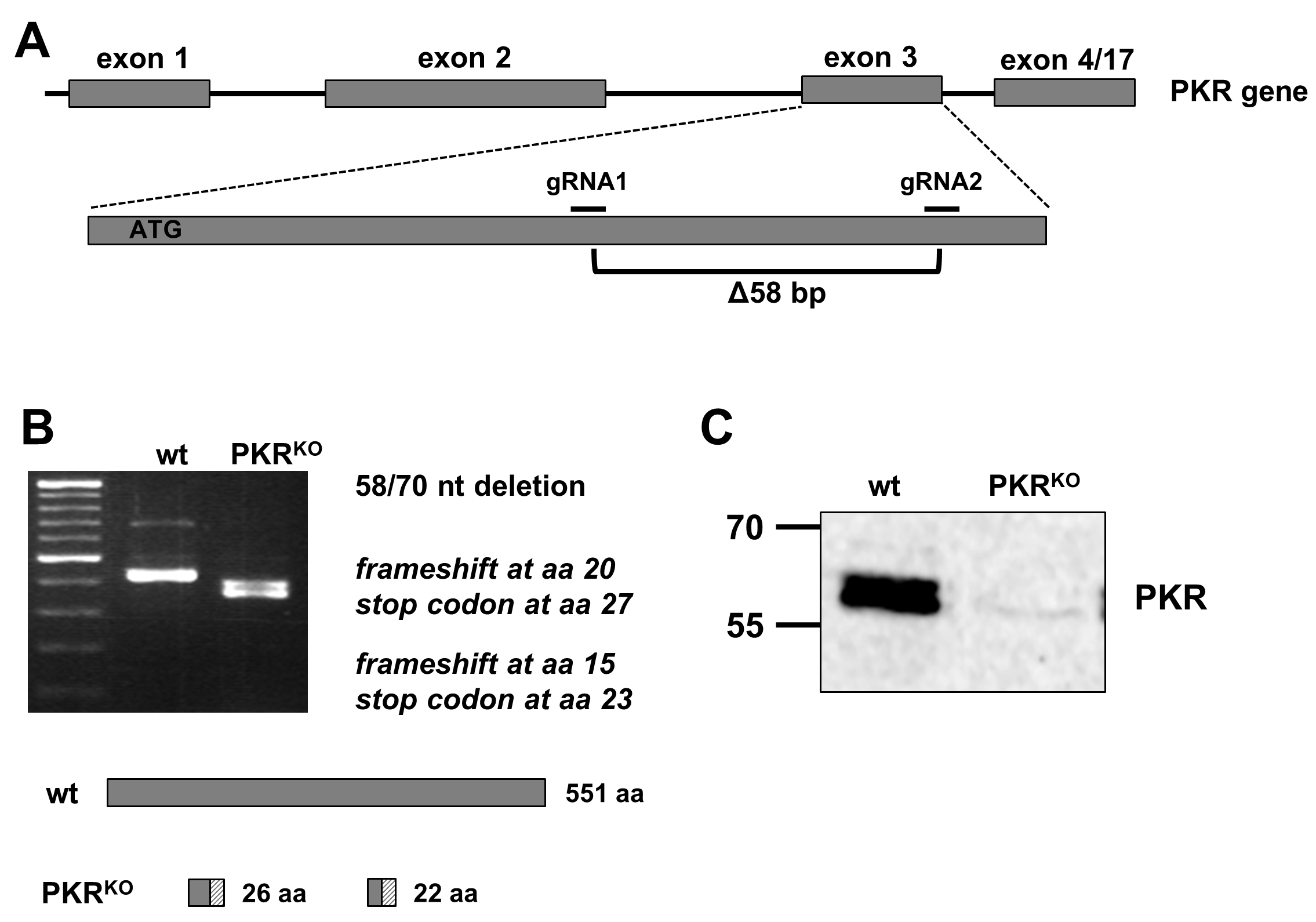

Supplement: S1 Fig — (A) Schematic representation of the PKR gene. Two guide RNAs were designed to target exon 3 of human PKR. (B) A single-cell clone was characterized by isolation of genomic DNA and integrity of human PKR gene was determined by sequence analysis. Both alleles contain a deletion resulting in a frame-shift event and a premature stop codon. (C) Western blot analysis of PKR protein levels in cell lysates from HeLa-wt or HeLa-PKRKO cells. (TIF) [file ppat.1005982.s001.tif]

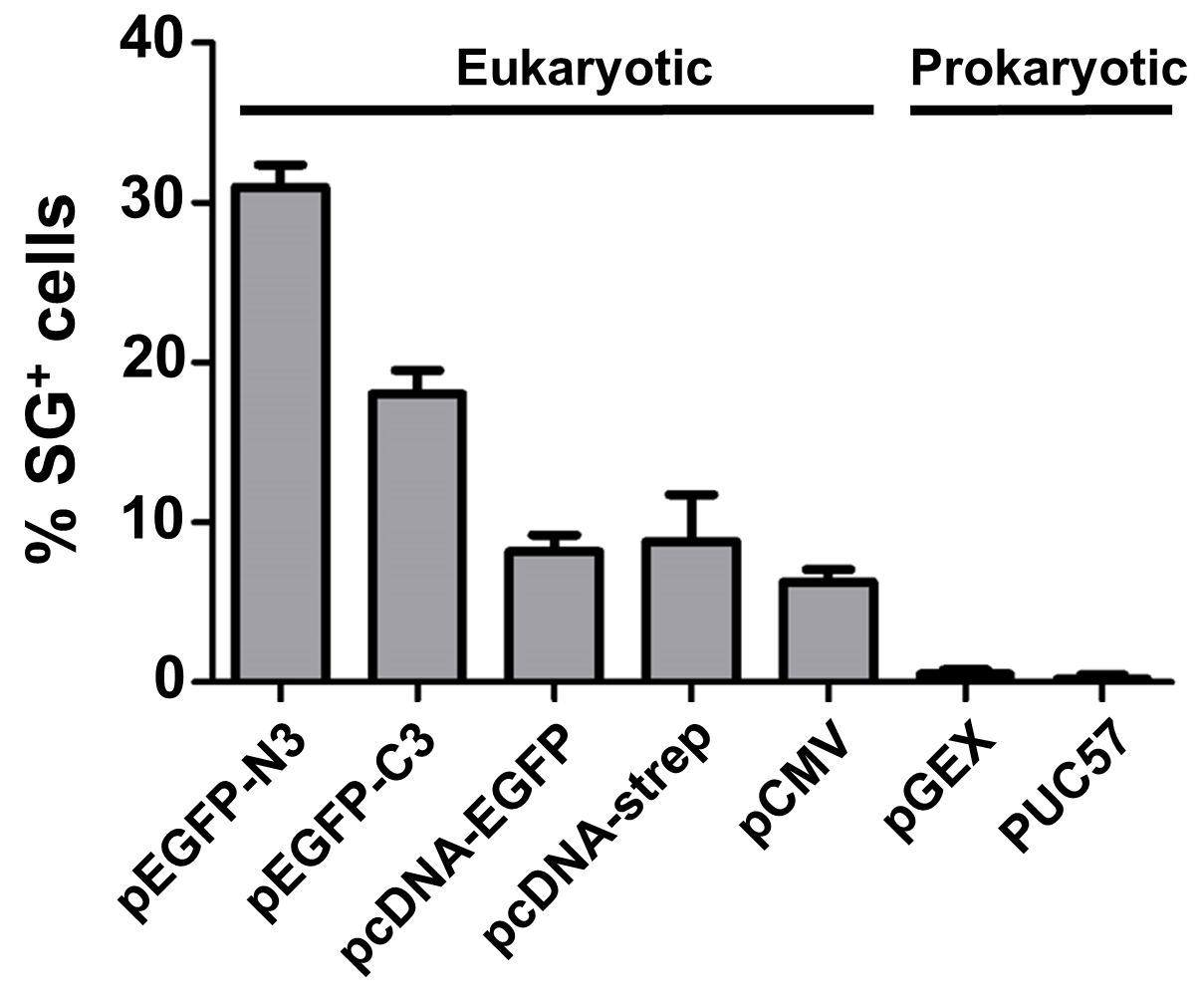

Supplement: S2 Fig — HeLa cells were transfected with different plasmids (500 ng/well). At 24h post transfection, cells were fixed and IFA was used to quantify the level of cells that possess SGs. For each type of plasmid, SG-positive cells were quantified from three randomly selected images and depicted in a bar-graph. (TIF) [file ppat.1005982.s002.tif]

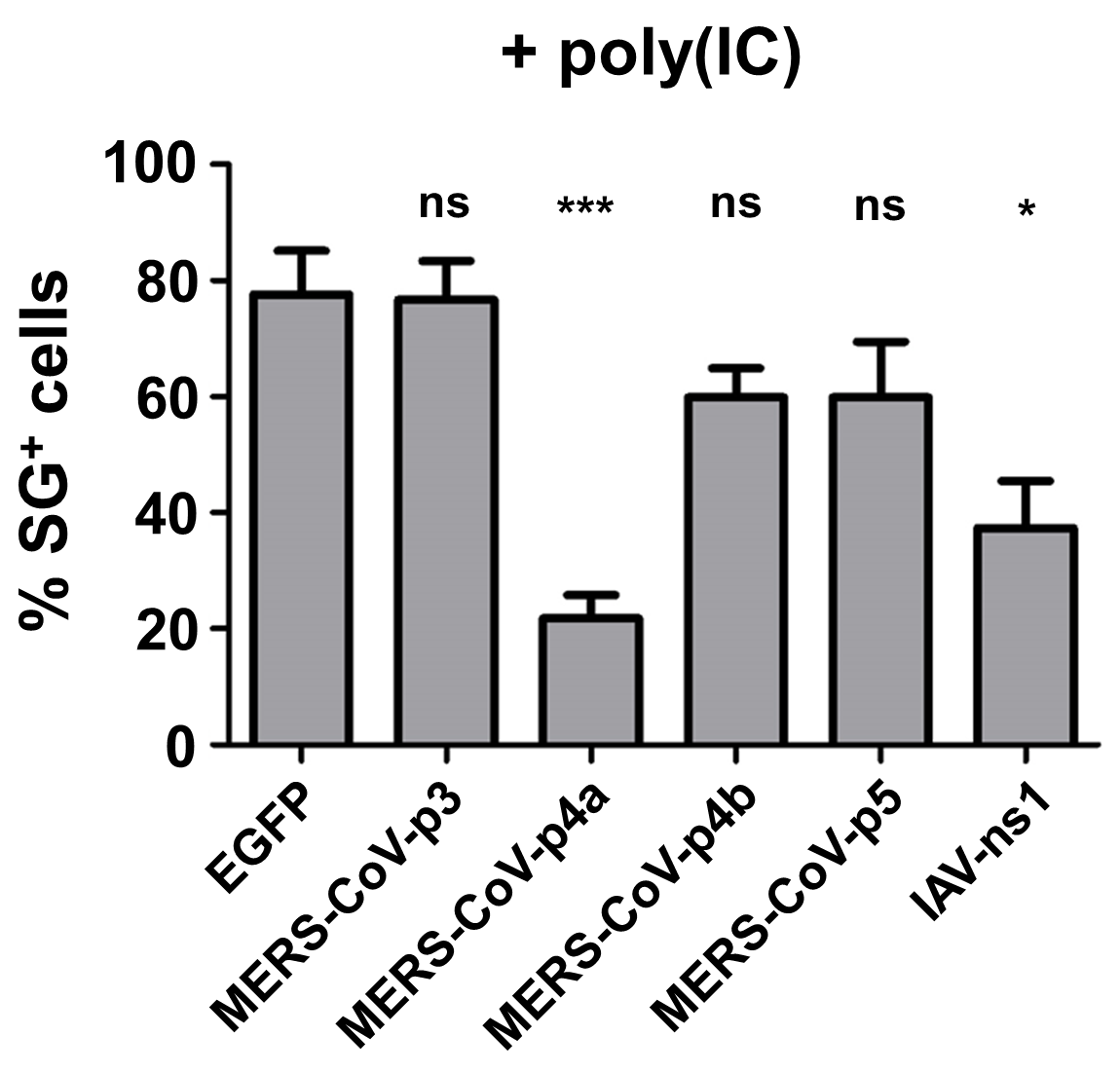

Supplement: S3 Fig — HeLa-wt cells were transfected with pEGFP-expression plasmids. Next day, SG formation was triggered by poly(I:C) transfection (100 ng/well). Cells were fixed using paraformaldehyde at 6h post RNA ligand transfection and SG formation was visualized using IFA. Quantification of SG-positive cells is shown as means and standard deviations of at least three randomly selected images per sample. Data was analyzed using an unpaired t-test (***, p<0.001; *, p<0.05; ns, not significant). (TIF) [file ppat.1005982.s003.tif]

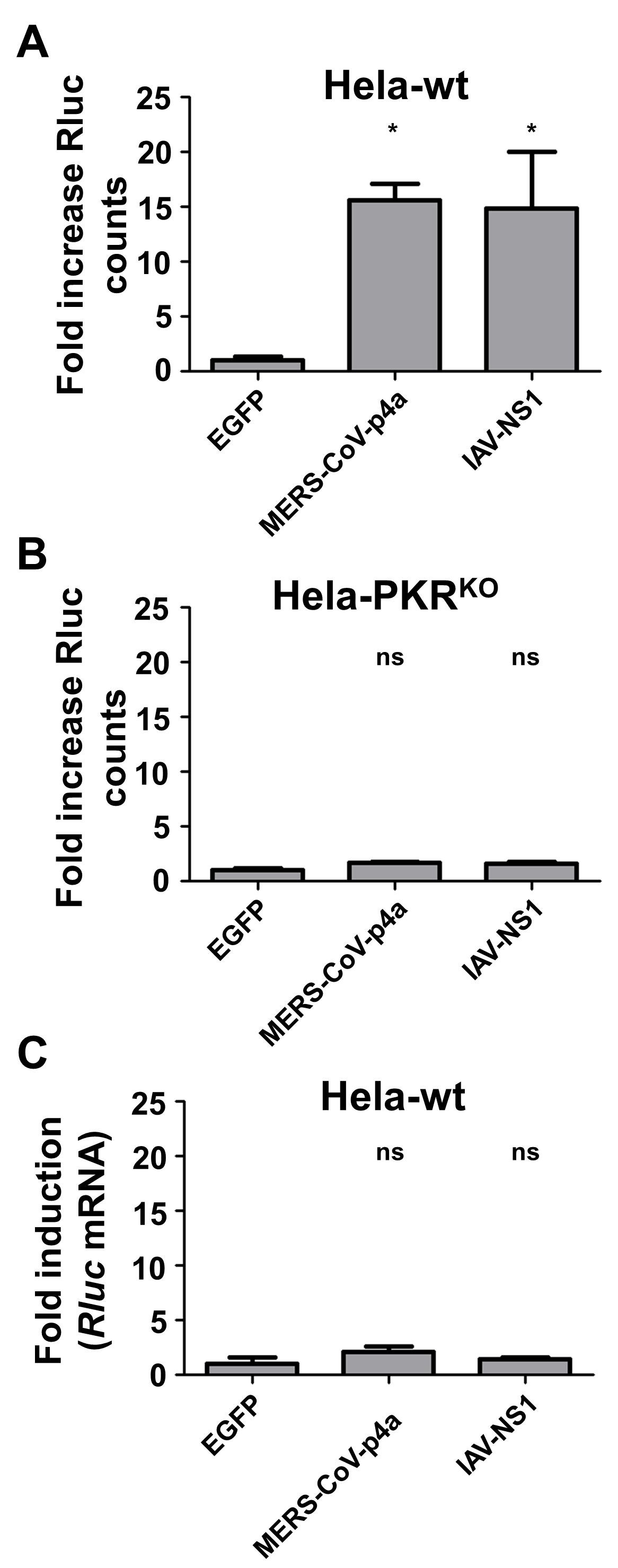

Supplement: S4 Fig — (A, B) Relative luciferase mRNA (A) and protein (B) levels in HeLa-wt cells co-transfected with pTK-RLuc and pEGFP expression plasmids. (C) Relative luciferase counts measured at 16 h post co-transfection of pTK-RLuc and pEGFP expression plasmids in Hela-PKRKO (D) cells. Data was analyzed using an unpaired t-test (***, p<0.001; *, p<0.05; ns, not significant). (TIF) [file ppat.1005982.s004.tif]

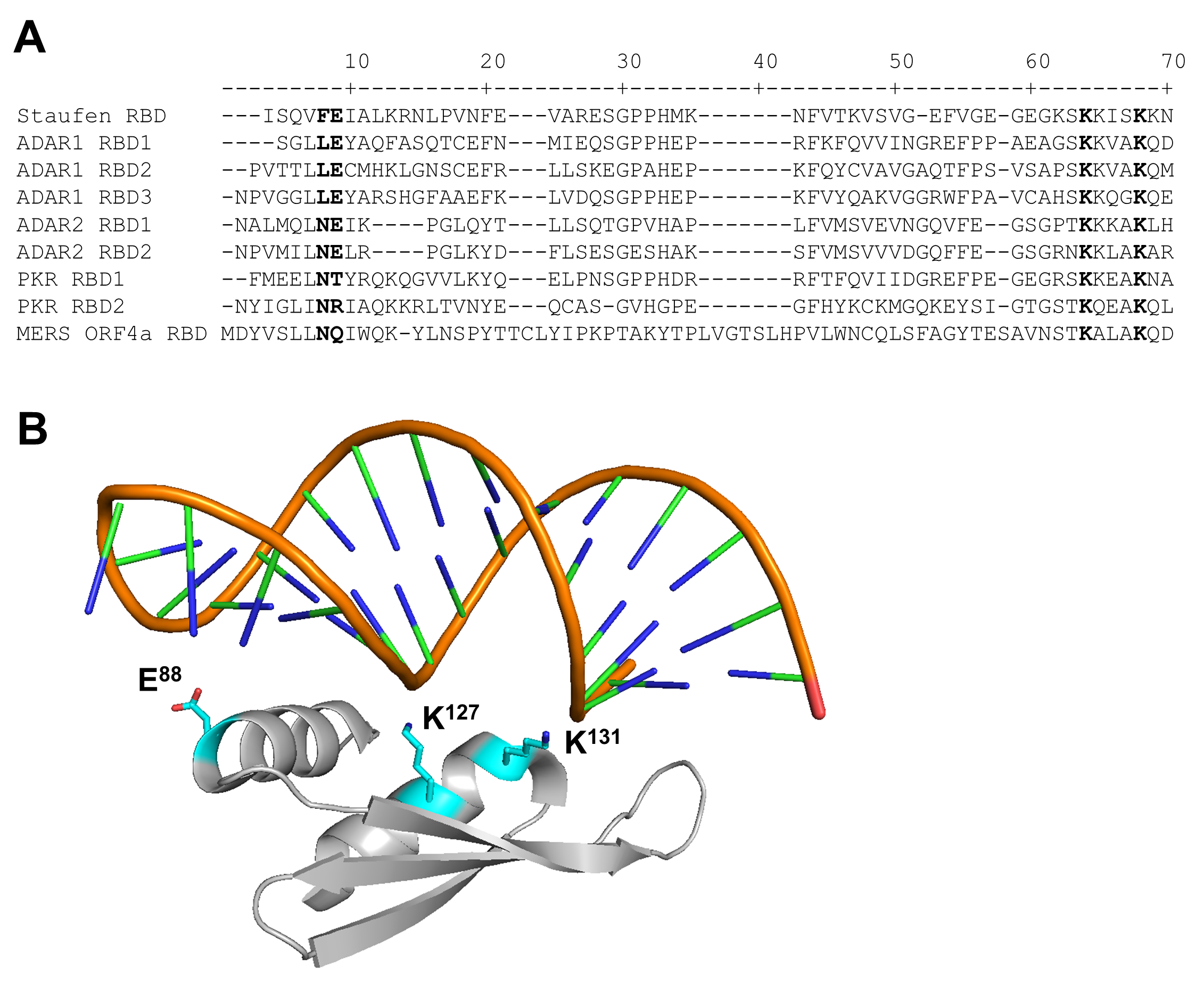

Supplement: S5 Fig — (A) Alignment of MERS-CoV p4a with other dsRNA binding motifs of several cellular proteins. In bold are the conserved residues crucial for dsRNA binding. (B) Structure of ADAR1 dsRNA binding motif in association with dsRNA. Highlighted are the corresponding ADAR1 residues that are mutated in MERS-CoV p4a in this study. (TIF) [file ppat.1005982.s005.tif]
